# Supplementary material for: Length of stay and economic sustainability of virtual ward care in a medium-sized hospital of the UK: a retrospective longitudinal study
Source: BMJ Open. 2024 Jan 24;14(1):e081378. doi: 10.1136/bmjopen-2023-081378 (PMC10823930; doi:10.1136/bmjopen-2023-081378)
Supplement: Supplementary data [file bmjopen-2023-081378supp001.pdf]

Length of stay and economical sustainability of virtual ward care in a medium sized hospital of the UK. A retrospective longitudinal study

Abdollah Jalilian[1,*], Luigi Sedda[1,*,$], Alison Unsworth[2] and Martin Farrier[2,#]

Supplementary information

Supplemental Table S1. Comparison of matching health indicators between all 366 patients admitted to virtual wards and the 318 virtual ward patients who were matched with patients admitted exclusively to the hospital. Based on t-tests for equality of means and chi-square tests for equality of proportions, no significant differences were found between the two groups at the significance level of 5%.

	Matched virtual ward patients (318)	All virtual ward patients (318+48)
Average CFS score	3.97	3.99
Average age	67.7	67.97
Average length of stay in virtual wards (from virtual wards onboarding to virtual wards offboarding)	9.79 days	9.64 days
Total number of virtual ward days	3114	3508
Number (percentage) of female patients	174 (54.7%)	203 (55.5%)
Number (percentage) of patients with diabetes	47 (14.8%)	54 (14.6%)
Number (percentage) of patients with hypertension	103 (32.4%)	125 (34.2%)
Number (percentage) of patients with chronic heart disease	36 (11.3%)	45 (12.3%)
Number (percentage) of patients with chronic kidney disease	2 (0.6%)	7 (1.9%)
Number (percentage) of patients with chronic obstructive pulmonary disease	81 (25.5%)	90 (24.6%)
Number (percentage) of patients with asthma	8 (2.5%)	14 (3.8%)

Jalilian A, et al. BMJ Open 2024; 14:e081378. doi: 10.1136/bmjopen-2023-081378

Supplemental Table S2. Costs for the provision of virtual ward for the calendar year of 2022.

Item	Whole Time Equivalent	Cost £
Nurses (band 5 and above)	12.5	520,032
Healthcare Assistant / Ancillary / Admin (Band 4 and below)	6.8	189,538
Pharmacists	2	122,239
Consultants	0.5	46,709
Corporate non clinical	1.45	148,165
Hardware		5,874
Travel		935
Medical Equipment		13,529
Additional Costs		4,129
Total		1,051,150

Jalilian A, *et al. BMJ Open* 2024; 14:e081378. doi: 10.1136/bmjopen-2023-081378
