# Supplementary material for: Length of stay and economic sustainability of virtual ward care in a medium-sized hospital of the UK: a retrospective longitudinal study
Source: BMJ Open. 2024 Jan 24;14(1):e081378. doi: 10.1136/bmjopen-2023-081378 (PMC10823930; doi:10.1136/bmjopen-2023-081378)
Supplement: Supplementary data [file bmjopen-2023-081378supp002.pdf]

STROBE checklist compliance

Strobe Statement

Title and abstract

1a and b Compliant

Introduction

2 and 3 Compliant

Methods

4 and 5 Compliant

6 As far as is possible this is explained in the text. Matching criteria are included.

7 Compliant

8 Compliant. The sources of data are the same for each group.

9 Compliant. This is addressed in the text.

10 Compliant. This is addressed in the text. The study size was “all patients”

11Compliant. This is addressed in the text.

12 Compliant. This is addressed in the text

Participants

13 Compliant. There are 2 stages, the study period (2022) and a check on whether the patient is alive at 30th April 2021. All patients are considered at both stages. There is no additional value in a flow diagram.

14 Compliant. This is addressed in the text.

15 Compliant.

16 Compliant.

17 Compliant.

Discussion

18 Compliant.

19 Compliant.

20 Compliant.

21 Compliant.

Other information

22 DOI completed for each of the authors. No funding has been received for this work.
