# Supplementary material for: Length of stay and economic sustainability of virtual ward care in a medium-sized hospital of the UK: a retrospective longitudinal study
Source: BMJ Open. 2024 Jan 24;14(1):e081378. doi: 10.1136/bmjopen-2023-081378 (PMC10823930; doi:10.1136/bmjopen-2023-081378)
Supplement: Author's manuscript [file bmjopen-2023-081378.draft_revisions.pdf]

BMJ Open is committed to open peer review. As part of this commitment we make the peer review history of every article we publish publicly available.

When an article is published we post the peer reviewers' comments and the authors' responses online. We also post the versions of the paper that were used during peer review. These are the versions that the peer review comments apply to.

The versions of the paper that follow are the versions that were submitted during the peer review process. They are not the versions of record or the final published versions. They should not be cited or distributed as the published version of this manuscript.

BMJ Open is an open access journal and the full, final, typeset and author-corrected version of record of the manuscript is available on our site with no access controls, subscription charges or pay-per-view fees (http://bmjopen.bmj.com).

If you have any questions on BMJ Open's open peer review process please email info.bmjopen@bmj.com

BMJ Open

Length of stay and economical sustainability of virtual ward care in a medium sized hospital of the UK. A retrospective case-control study.

Journal:	*BMJ Open*
Manuscript ID	bmjopen-2023-081378
Article Type:	Original research
Date Submitted by the Author:	26-Oct-2023

Complete List of Authors:	Jalilian, Abdollah; Lancaster University<br>Sedda , Luigi ; Lancaster University<br>Unsworth, Alison; Wrightington Wigan and Leigh NHS Foundation Trust<br>Farrier, Martin; Wrightington Wigan and Leigh NHS Foundation Trust, Paediatrics

Keywords:	Telemedicine < BIOTECHNOLOGY & BIOINFORMATICS, EPIDEMIOLOGIC STUDIES, HEALTH ECONOMICS, Health Services

SCHOLARONE™
Manuscripts

I, the Submitting Author has the right to grant and does grant on behalf of all authors of the Work (as defined in the below author licence), an exclusive licence and/or a non-exclusive licence for contributions from authors who are: i) UK Crown employees; ii) where BMJ has agreed a CC-BY licence shall apply, and/or iii) in accordance with the terms applicable for US Federal Government officers or employees acting as part of their official duties; on a worldwide, perpetual, irrevocable, royalty-free basis to BMJ Publishing Group Ltd ("BMJ") its licensees and where the relevant Journal is co-owned by BMJ to the co-owners of the Journal, to publish the Work in this journal and any other BMJ products and to exploit all rights, as set out in our licence.

The Submitting Author accepts and understands that any supply made under these terms is made by BMJ to the Submitting Author unless you are acting as an employee on behalf of your employer or a postgraduate student of an affiliated institution which is paying any applicable article publishing charge ("APC") for Open Access articles. Where the Submitting Author wishes to make the Work available on an Open Access basis (and intends to pay the relevant APC), the terms of reuse of such Open Access shall be governed by a Creative Commons licence – details of these licences and which Creative Commons licence will apply to this Work are set out in our licence referred to above.

Other than as permitted in any relevant BMJ Author's Self Archiving Policies, I confirm this Work has not been accepted for publication elsewhere, is not being considered for publication elsewhere and does not duplicate material already published. I confirm all authors consent to publication of this Work and authorise the granting of this licence.

For peer review only

Length of stay and economical sustainability of virtual ward care in a medium sized hospital of the UK. A retrospective case-control study

Abdollah Jalilian[1,*], Luigi Sedda[1,*,$], Alison Unsworth[2] and Martin Farrier[2,#]

[1]Lancaster Ecology and Epidemiology Group, Lancaster Medical School, Lancaster University, Lancaster, UK

[2]Wrightington Wigan and Leigh Teaching Hospitals NHS Foundation Trust, Wigan, UK

[*]These authors contributed equally to the work.

[$]Corresponding author: l.sedda@lancaster.ac.uk

[#]Senior author

For peer review only

Abstract

Objective

To evaluate length of stay difference and its economic implications between hospital patients and virtual ward patients.

Design

Retrospective case-control study.

Setting

Wrightington, Wigan and Leigh Teaching Hospitals National Health Service (NHS) Foundation Trust (WWL), a medium-sized NHS Trust in north-west of England.

Participants

Virtual ward patients (318 cases) were matched 1:1 to 1:4, depending on matching characteristics, to all hospital patients (350 controls). All patients were admitted to hospital during the calendar year of 2022.

Outcome measures

The primary outcome is the length of stay as defined from the date of hospital admission to the date of discharge/death (hospital patients) and from the date of hospital admission to the date of admission in virtual ward (virtual ward patients). The secondary outcome is the cost of a hospital bed day and the equivalent value of virtual ward saving in hospital bed days. Additional measures were 6 months readmission rates and survival rates at the follow up date of 30th April 2023.

Risk factors

Age, sex, comorbidities and clinical frailty score were used to evaluate the importance and effect of these factors on the main and secondary outcome.

Methods

Statistical analyses included logistic and binomial mixed models for length of stay in hospital and readmission rate outcomes, and Cox proportional hazard model for the survival of the patients.

Results

The virtual ward patients had a shorter stay in hospital before being admitted to the virtual ward (2.89 days, 95% CI 2.1-3.9 days). Chronic kidney disease and frailty were associated to longer length of stay in hospital (58% 95% CI 22%-100% compared to patients without CKD and 14% 95%CI 8%-21% compared to patients with one unit lower CFS). The frailty score was also associated to higher rate of readmission within 6 months and lower survival. Being admitted to virtual ward slightly improved survival, although when readmitted the survival deteriorated rapidly. The cost of a 24 hour period in a general hospital bed is £536. The cost of a day hospital saved by virtual ward was £935.

Conclusion

The use of a 40 virtual bedded ward was clinically effective in terms of survival for patients not needing readmission and allowed for the freeing of 3 hospital beds per day. However, the cost for each day freed from hospital stay was three quarters larger than the one for a single day hospital bed. This raises concerns on the deployment of large scale virtual wards without the existence of policies and plans for their cost-effective management.

For peer review only

For peer review only - http://bmjopen.bmj.com/site/about/guidelines.xhtml

What is already known on this topic

There are several virtual wards experiences around the world, most of them created to reduce hospital pressure from COVID-19 patients or for post-recovery management.

All the UK-based studies are based on a relatively small number of virtual wards patients with virtual wards designed to specific conditions or diseases.

None of the UK-based study produced a cost-effectiveness of virtual wards.

While in general virtual wards seem to reduce the length of stay in hospital, other clinical outcomes, such as readmission and survival results are conflicting.

What this study adds

In order for the virtual wards to be cost-effective they need to almost halve their costs without affecting patients' clinical outcomes. However, the higher rate of readmission to hospital from virtual wards patients, compared to hospital patients, can hamper any gain from reduction in costs, highlighting the necessity of appropriate plans and procedure for the selection, design and management of the virtual wards and their patients.

This evidence should be taken into consideration by NHS in planning the next large deployment of virtual wards within the UK.

Strengths and limitations

1. This is the largest virtual wards study, in terms of virtual wards patients, and the first in UK assessing their cost.
2. Patients are matched for an extensive number of characteristics and most of the virtual ward patients are used for this analysis. In addition, some virtual ward patients were matched with more than one hospital patient to control for all hospital inpatient heterogeneity.
3. Within 40% of the virtual ward patients, there is only a single pair of matched patients with a specific primary diagnosis description, raising concerns about potentially insufficient data availability for certain conditions. The lack of randomisation due to the relatively small sample size can introduce potential residual confounding and biases. This can limit the generalisability of the results and warrant further investigation.
4. It was not possible to ascertain the inclusion and exclusion criteria used to send patients to virtual wards. However, matching was still possible for several baseline characteristics reducing the risk of bias.
5. Exact costs for hospital patients were not available for this research, but this figure (£536) is coherent with the cost provided in other studies [24]. In addition, calculations relative to the increasing of the number of virtual wards does not account for potential economy of scale effects.

For peer review only - http://bmjopen.bmj.com/site/about/guidelines.xhtml

Introduction

Virtual wards offer patients the opportunity to receive health care in the comfort and safety of their own homes, including care homes [1]. Pioneered in the UK, virtual wards became a popular scheme during COVID-19 [2] and since then it has been replicated in other 'thematic virtual wards' (i.e. respiratory, heart failure and chronic obstructive pulmonary disease to name few). Virtual wards are the response to the chronic and unsustainable high demand for secondary care beds [3]. By providing an alternative to continued admission, virtual wards may help bridge the gap between demand and capacity for hospital beds especially in the context of aging population [3 4], although it' successes are still debated (see for example [5] review on randomised controlled trials of virtual wards for acute respiratory infection). A plan to enrol virtual wards in most of the Integrated Care Systems in UK has been launched in 2020 with the aim to make available hundreds of thousands of virtual beds (at a cost of hundreds of million pounds) in the next years in order to provide them as an option for every patient [6].

Virtual Wards must be cost effective if they are to replace traditional inpatient care, the costs must be comparable or lower than the costs of hospital stay to be economically sustainable in the medium to long terms. To establish whether large scale implementation of virtual wards could alter the provision of healthcare, it is necessary therefore to address workforce issues and reduce the cost of modern healthcare.

The main objective of this research is to compare the length of stay in the hospital for hospital and virtual wards admitted patients. We define the length of stay as the duration, in days, between the date of admission to the hospital and the date of discharge from the hospital for hospital patients, and from date of admission to the hospital and the date of admission to virtual ward for virtual ward patients. By analysing the length of stay, we aim to assess the effectiveness of virtual wards in reducing the duration of hospitalization and therefore increasing the number of beds available for hospital patients. This is what is also defined as 'step-down' model by [3].

Beyond potential reductions in the length of a patient stay in hospital, we aim to compare the survival outcomes of patients in both virtual wards and hospital settings. Additionally, we compare the readmission rates to the hospital within six months of discharge. Survival and readmission are two indicators of the clinical effectiveness of the virtual ward to satisfy the healthcare needs of the patients [7] .

By considering all these aspects—length of stay, survival, readmission rates and costs, it will be possible to assess the impact of virtual wards on patient care, and its effectiveness in comparison to traditional inpatient care. The findings from this study respond to recent requests on evidence-based informed virtual wards provision [8], and will contribute to a deeper understanding of the potential benefits and implications of virtual wards as an alternative care model.

For peer review only - http://bmjopen.bmj.com/site/about/guidelines.xhtml

Material and methods

Data

The study follows a retrospective case-control design where cases are patients designated for virtual wards who undergo a partial discharge process from the hospital and continue to receive medical attention and support within their homes until they complete their treatment in the Virtual Ward setting and are then fully discharged; and controls who are patients that receive care within the hospital until they are fit for full discharge.

Cases are matched manually with patients admitted exclusively to the hospital, resulting in 350 case-control pairs. Patient Administration System data was used to pull a list of age matched patients who were admitted during the same study year. Further data from coding was then used to provide full matching for cases. These patient pairs comprised 350 unique hospital patients and 318 virtual ward patients. Therefore 291 are 1 case to 1 control matched; 23 are 1:2; 3 are 1:3 and finally 1 is 1:4. The study group comprises all the patients admitted to Wrightington, Wigan and Leigh Teaching Hospitals NHS Foundation (WWL) during the calendar year 2022 (1st of January 2022 to the 31st of December 2022) and their progress was followed up on the 30th of April 2023.

Matching characteristics were sex, age, primary diagnosis description and Clinical Frailty Score (CFS, 1 representing a very fit patient to 9 indicating a terminally ill patient). In particular, all matched patients have the same gender and the same primary diagnosis description. 97% of the matched patients possess the same CFS score, while the remaining 3% have a CFS scores within a range of ±2 points. Finally, 95% of the matched patients share the same age or have an age difference within ±6 years, with the remaining 5% having an age gap of less than 12 years. Matching was exact where possible but for a minority of patients an exact match was not possible in the dataset for the study year. In that case the closest possible match was achieved leading to the ranges in CFS and age.

Additional unmatched information was provided for each patient: comorbidities, readmission information, date of admission and discharge to and from hospital, date of admission and discharge to and from virtual ward and date of death when applicable up to 30th April 2023. Due to the study design and rigorous data entry checks, there is no missing data.

48 patients admitted to virtual wards did not have suitable matches among the patients admitted exclusively to the hospital during the study year. Consequently, these 48 patients were excluded from the analyses.

To estimate the cost-effectiveness of virtual ward admission compared to exclusively hospital ward admission the costs of facilities and staffing were included in the analysis. Financial data for the cost of the virtual ward was maintained separate from other Trust Finances so that the cost of the virtual ward could be fully understood. Financial data were provided by the WWL Trust Finance Team.

Patient and public involvement

Owing to use of retrospective data sources, no members of the public or patients were formally involved in the study's design, analysis, or manuscript writing, due to the limited size of the research group and the absence of funding. The communication plan includes executive summaries circulated at the NHS Trust, press releases, public lectures, and social media posts in order to reach the most diverse audience.

For peer review only - http://bmjopen.bmj.com/site/about/guidelines.xhtml

Methods

Study objectives and respective methods are summarized in Table 1. Because some virtual ward patients have been matched with more than one hospital patient, a random effect representing recurring virtual ward patients is included in the models.

Table 1. Statistical analyses employed for the four study outcomes.

	Primary outcome	Secondary outcome	Additional outcomes	
	Length of stay (in days)	Bed cost	Being readmitted to the hospital within 6 months after discharge [9]	Survival at the follow up date of 30/4/2023
**Question**	Which are the factors influencing the length of stay in the hospital?	What is the daily cost of a bed in hospital and a bed in virtual ward?	What are the factors associated to readmission of patients within 6 months after discharge?	What are the factors contributing to the survival of patients?
**Statistical Model**	Generalized mixed model with generalized Poisson response	Descriptive approach: direct collation of cost for Virtual Ward compared with NHS approved methodology for non-elective inpatient bed cost at the same organisation.	Logistic regression model	Cox proportional hazard model

Models were run within a Bayesian framework with assigned non-informative prior distributions to all parameters. The computations were performed by using the R-INLA package [10] to allow to incorporate uncertainty in the data and estimate the posterior distributions of the model parameters using the INLA methodology [10].

Virtual ward cost is calculated from accrued expenditure. This was kept as a separate spreadsheet so that exact costs were known. The virtual ward utilizes 12.5 WTE nurses and 0.5 WTE Consultants with the assistance of 2 WTE pharmacists and 6.8 WTE ancillary staff members. NICE recommendations[a] provide a basis for the calculation of standard ward staffing for an idealized inpatient ward. Using their recommendations, a typical 28 bedded hospital ward would require 35 WTE nurses to staff with 12 WTE ancillary staff members. Medical staffing for a traditional 28 bedded ward would depend on the clinical work but would require a ward round each day and the involvement of a Consultant and Junior medical staff.

Cost estimation for hospital bed day uses NHS agreed methodology. It represents the average cost of non-elective care provision in wards on the Acute Hospital site. It is calculated by the Finance Department at the organisation and is in line with the costs submitted by the organisation to NHS England. This is because

[a] NICE https://www.nice.org.uk/guidance/sg1/resources/illustration-of-process-pdf-11945197

For peer review only - http://bmjopen.bmj.com/site/about/guidelines.xhtml

patients who are cared for in the virtual ward are by definition in the second half of their inpatient hospital stay. Costs are higher for the first day of a patient admission to hospital than they are for subsequent days because patients are likely to need more care, tests and intervention in the first part of their stay. At the moment an estimate of cost for the second half of a patients stay in hospital is not available, only the average cost for all days spent in hospital has been provided. As a consequence, the estimate of hospital stay is a high estimate and the virtual ward cost is an exact cost.

Results

Summative and descriptive analysis

The primary diagnosis description for the majority (64.3%) of the matched patients corresponds to the conditions pneumonia, chronic obstructive pulmonary disease (COPD), COVID-19, atrial fibrillation, or heart failure (HF). Overall, patients admitted to virtual wards exhibit a shorter hospital stay in comparison to their matched counterparts in the hospital (p-value < 0.001) (Table 2). Based on a 95% confidence interval, it is estimated that virtual ward patients experience a reduction in hospital stay ranging from 2.1 to 3.9 days compared to their corresponding matches in the hospital.

Table 2. Baseline demographics of cases and controls. Number of stars resulting from the p-value obtained from a paired t-test: * if 0.01 ≤ p-value < 0.05, ** if 0.001 ≤ p-value < 0.01 and *** if p-value < 0.001. No stars indicate a p-value above 0.05. For comorbidities, only those above 2% are shown in the table.

Baseline demographics	Patients admitted only to the hospital (controls = 350)	Patients admitted to virtual wards (cases = 318)	p-value
Percentage of female patients	54.6%	54.7%	
Median age (years)	72	71	
Median CFS score	4	4	
Number (percentage) of patients with Pneumonia as their primary diagnosis description	70 (20%)	61 (19.2%)	
Number (percentage) of patients with Chronic obstructive pulmonary disease as their primary diagnosis description	63 (18%)	55 (17.3%)	
Number (percentage) of patients with COVID as their primary diagnosis description	38 (10.9%)	33 (10.4%)	
Number (percentage) of patients with Atrial Fibrillation as their primary diagnosis description	30 (8.6%)	27 (8.5%)	
Number (percentage) of patients with Heart Failure as their primary diagnosis description	24 (6.9%)	22 (6.9%)	
Number (percentage) of patients with Hypertension as their primary diagnosis description	15 (4.3%)	14 (4.4%)	
Number (percentage) of patients with Cellulitis as their primary diagnosis description	11 (3.1%)	11 (3.4%)	
Number (percentage) of patients with Chest infection as their primary diagnosis description	10 (2.9%)	10 (3.1%)	
Average length of stay in the hospital (from hospital admission to hospital discharge or to virtual ward)	5.96 days	2.89 days	***

For peer review only - http://bmjopen.bmj.com/site/about/guidelines.xhtml

Average length of stay in virtual wards (from virtual wards onboarding to virtual wards offboarding)	---	9.79 days	NA
Total number of hospital days used by patients	2086	920	
Total number of virtual wards days used by patients	---	3114	
Average length of care in both hospital and virtual wards	5.96 days	12.96 days	***
Number (percentage) of patients with diabetes	86 (24.6%)	47 (14.8%)	**
Number (percentage) of patients with hypertension	159 (45.4%)	103 (32.4%)	***
Number (percentage) of patients with chronic heart disease	123 (35.1%)	36 (11.3%)	***
Number (percentage) of patients with chronic kidney disease	51 (14.6%)	2 (0.6%)	***
Number (percentage) of patients with chronic obstructive pulmonary disease	128 (36.6%)	81 (25.5%)	**
Number (percentage) of patients with asthma	39 (11.1%)	8 (2.5%)	***
Number (percentage) of patients with learning disability	5 (1.4%)	1 (0.3%)	
Number (percentage) of patients being readmitted to the hospital within 6 months after discharge	108 (30.9%)	128 (40.3%)	*
Number (percentage) of patients being alive on the follow up date 30/4/2023	275 (72.4%)	275 (86.5%)	*

In general, patients admitted to virtual wards have lower prevalence rates of asthma, HF, chronic kidney disease (CKD), COPD, diabetes, hypertension, and learning disability compared to the patients staying in hospital. Moreover, a slightly higher percentage of patients admitted to virtual wards were alive on the follow-up date in comparison to patients admitted exclusively to the hospital (although bordering statistical significance). Finally, readmission rate within six months after discharge is higher for patients admitted to virtual wards than hospital patients. Only one patient was re-admitted to hospital during the virtual ward clinic.

Virtual wards patients used to match hospital patients were representative of the total virtual ward patient cohort (Supplemental Table S1).

Length of stay in hospital, readmission and survival models.

The results of the logistic regression model to the binary variable of being readmitted within 6 months of discharge (summarised in Table 3) indicates that several factors are significantly influencing the likelihood of readmission within 6 months of discharge. Older age and higher CFS score, and having COPD, are associated with increased chances of readmission. Specifically, for every one-unit increase in age, while keeping all other factors at their average level, there is a 3% increase in the odds of readmission. Similarly, for each unit increase in the CFS score, while holding all other factors at their average level, the odds of readmission increases by 20%. Having a diagnosis of COPD leads to a 70% higher odds of readmission. Notably, being admitted to virtual wards is the most substantial risk factor for readmission within the next 6 months. Virtual ward patients face a significant 120% increased odds of readmission compared to their counterparts in the hospital.

Table 3. Odds ratios and their corresponding 95% credible intervals for factors affecting the probability of being readmitted within 6 months of attendance (in bold the statistically significant factors at a significance level of 5%).

	odds ratio	95% credible interval	
Intercept	0.033	0.01	0.083

For peer review only - http://bmjopen.bmj.com/site/about/guidelines.xhtml

Gender Male	0.756	0.531	1.044
**Age**	**1.027**	**1.011**	**1.043**
**CFS score**	**1.176**	**1.035**	**1.329**
Diabetes	1.441	0.924	2.146
Hypertension	0.732	0.508	1.022
Heart failure	0.955	0.61	1.424
Chronic kidney disease	1.408	0.7	2.54
**Chronic obstructive pulmonary disease**	**1.673**	**1.148**	**2.356**
Asthma	0.949	0.428	1.833
Learning disability	9.104	0.937	36.551
Length of stay	1.023	0.997	1.048
**Admitted to virtual wards**	**2.226**	**1.506**	**3.172**

The results from the Cox proportional hazard model applied to the survival from admission to either death or being alive at the follow up date are shown in Table 4. The proportional hazards assumption for the fitted Cox model was validated (p-value = 0.11), indicating that the hazards were proportional over time. Based on these results the following conclusions can be made:

1. Patients admitted to virtual ward had a significant lower mortality (63%) compared to those admitted to hospital.
2. Patients with heart failure show a 75% higher risk of death compared to those without this condition.
3. For each unit increase in the CFS score, while holding all other factors at their average level, the risk of death increases by approximately 35%.
4. There is a 4% increase in the risk of death for each additional day of hospital stay, while keeping all other factors at their average level.
5. Patients who are readmitted within 6 months of discharge face a significant increase in the risk of death. Specifically, virtual wards patients readmitted in hospital experience a 220% higher risk of dying compared to the 175% of hospital patients readmitted to hospital when compared to non-readmission patients.

Table 4. Hazard ratios and their corresponding 95% credible intervals for factors affecting the death of patients from the date of admission (in bold the statistically significant factors at a significance level of 5%).

	hazard ratio	95% credible interval	
Intercept	0.001	0	0.004
Gender Male	1.03	0.703	1.457
Age	1.009	0.99	1.028
**CFS score**	**1.351**	**1.181**	**1.538**
Diabetes	0.966	0.605	1.465
Hypertension	0.915	0.615	1.311
**Hearth Failure**	**1.762**	**1.143**	**2.597**
Chronic kidney disease	1.047	0.556	1.8
Chronic obstructive pulmonary disease	1.376	0.937	1.951
Asthma	0.405	0.104	1.097

For peer review only - http://bmjopen.bmj.com/site/about/guidelines.xhtml

Learning disability	0.867	0.065	3.843
**Admitted to virtual ward**	**0.370**	**0.160**	**0.750**
**Length of stay**	**1.04**	**1.02**	**1.06**
**Readmission 6 months: hospital patient**	**2.763**	**1.688**	**4.272**
**Readmission 6 months: Admitted to virtual wards**	**3.179**	**2**	**4.805**

Table 5: Risks and their corresponding 95% credible intervals for factors associated to the length of stay in hospital of patients (in bold the statistically significant factors at a significance level of 5%).

	Exp(coefficients)	95% credible interval	
Intercept	4.116	2.566	6.29
Gender Male	0.938	0.796	1.099
Age	0.995	0.988	1.002
**CFS score**	**1.144**	**1.077**	**1.213**
Diabetes	1.103	0.91	1.323
Hypertension	1.035	0.874	1.217
Hearth Failure	1.134	0.918	1.383
**Chronic kidney disease**	**1.579**	**1.224**	**2.004**
Chronic obstructive pulmonary disease	1.077	0.872	1.315
Asthma	1.163	0.867	1.527
Learning disability	1.287	0.651	2.29
Readmission within 6 Months	1.084	0.918	1.271
**Admitting to virtual wards**	**0.563**	**0.47**	**0.669**

Finally, the length of stay is associated to several factors (Table 5):

1. Having CKD is associated with an average increase of around 60% in the length of stay.
2. For each unit increase in the CFS score, while holding all other factors at their average level, there is an average increase of around 15% in the length of stay.
3. Being admitted to virtual wards, as opposed to staying in the hospital, results in an average decrease of around 45% in the length of stay in hospital before admission to virtual ward.

Including an interaction term for each co-morbidity and hospital treatment (virtual ward or inpatient hospital), does not produce statistically significant coefficients for any of these terms.

Hospital and virtual ward costs

The costs for the virtual ward during the Calendar Year 2022 are shown in Supplemental Table S2. The total was around £1.051 million for 40 virtual ward beds. There were additional costs of £100,000 as part of the process of setting up the service, but these are non-recurrent costs and therefore not included in the final costings for the virtual ward.

The calculated reduction of in-patient hospital bed days for patients admitted to the virtual ward was 3.07 days for each of the patients admitted to the virtual ward (Table 2, 5.96 vs 2.89 days). There were 366 patients admitted to the virtual ward during the study period for which the costs of the virtual ward were £1.051

For peer review only

million. Using those figures, the cost of saving one in-patient hospital day was £935 (£1.051.150/(3.07 days x 366 patients)).

The average cost of a non-elective in-patient bed day within the organisation using standard NHS methodology was provided by the WWL Finance Department and amounted to £536.

Comparison of costs for the virtual ward with the costs of a continued inpatient stay is therefore possible. The cost of reducing a patients hospital stay by one day is £935, when they are admitted to the virtual ward as a strategy for shortening their in-patient stay. If the patient had stayed in hospital rather than being admitted to the virtual ward, the equivalent in-patient hospital day cost would have been £536. Therefore, the cost of virtual ward care is approximately three quarters higher than that of traditional in-patient care. However, the £935 are calculated based on the WWL capacity to use the virtual beds, which was 24% of the potential 14600 24 hour beds per year provided by 40 virtual wards beds (Supplemental Table S1, reference to 3508 total days spent by virtual ward patients).

For peer review only

For peer review only - http://bmjopen.bmj.com/site/about/guidelines.xhtml

Discussion

This study has shown that virtual ward patients had a shorter length of stay in hospital, but more likely to be re-admitted in hospital, and when re-admitted have a lower chance to survive compared to non-readmitted patients and readmitted patients previously discharged from hospital. Virtual ward patients were affected by a lower number of comorbidities, although their frailty scores along with sex, age and primary diagnosis were matched with hospital patients. Whilst virtual wards are shown to effectively reduce the length in-patient stay, its cost associated to a freed day hospital bed is three quarters higher than that of in-patient hospital care.

Previous studies have focused on the development of virtual wards for specific conditions or group of patients. For the UK, one of the first countries that trialled virtual wards, literature is scarce and the size of virtual wards are smaller compared to the one presented here [3 11 12]. In addition, this study is the first example of the assessment of virtual ward cost effectiveness in UK within a medium size hospital facility.

In terms of virtual ward clinical outcomes, current knowledge provides conflicting evidence [13] apart from randomised controlled trails enrolling patients with heart failure [7]. Although a direct comparison of the present study with current literature over the clinical outcomes is not possible, some common patterns can be highlighted.

As in other studies, patients admitted to the virtual ward had lower rates of comorbidity (see for example the review for virtual ward COVID-19 by [14]). While recent literature reviews found lower rate of re-admission in COPD older patients [3] and COVID-19 patients [15] after hospital at home and virtual wards, the present study did not find any significant difference as also reported in [5]. However, readmission rates for all patients (hospital and virtual wards) increased if a patient was from a virtual ward, older and with COPD. The higher rate of readmission is coherent with clinical expectations. The care provided by the virtual ward is, in part, a monitoring process, therefore clinical deterioration is often followed by readmission of the patient [11]. For discharged hospital patients, readmission would need to be initiated by the patient. For those virtual ward patients not readmitted, the care is shown to be non-inferior, and other studies have indicated that care in virtual wards is preferred by patients and also by the healthcare staff [3 16-18].

Survival was better in patients admitted to the virtual ward but decreases in patients from virtual wards when readmitted, and for those with hearth failure. A similar result was found in other studies [3 9]. Although virtual ward patients were matched by sex, age, primary diagnosis description and CFS, they showed a lower frequency of co-morbidities than in the controls which may have contributed to larger survival in their first admission compared to hospital patients. It is likely that, despite the careful matching of patients, there is a selection bias towards fitter patients when patients are being selected for consideration of admission to the Virtual Ward. Greater independence and ability to communicate with Virtual Ward staff by videophone make patients better candidates for admission to the Virtual Ward.

Our analysis found a length of stay in hospital prior to virtual ward admission to be shorter than the one in other studies, although many of them were based on COVID-19 virtual wards or other disease-specific virtual wards [5 17 19-21]. A common result, however, is a shorter stay in hospital for virtual wards patients than hospital patients. The present study highlighted that apart from CKD, the reduction in length of stay is not related to any other primary diagnosis, although dependent on the assessed CFS. Length of stay increases in patients with higher CFS and/or with CKD.

It is important to note that CFS is detrimental for all the clinical outcomes considered in this analysis: length of stay, readmission and survival. Another study, although on an elderly cohort, shown similar association between CFS and mortality [22]. These findings may suggest that patients with lower CFS are those that may

For peer review only - http://bmjopen.bmj.com/site/about/guidelines.xhtml

benefit more from virtual wards in terms of cost-effectiveness and clinical outcome. Clinical experience indicates that virtual ward care requires a patient to have some self-caring ability. The more compromised that is, the less likely a virtual ward is able to provide appropriate levels of care.

With focus on the length of stay which is the key aspect for the target of increasing hospital bed provision by NHS in the UK [b], this retrospective case control study of a virtual ward through a whole calendar year, provides evidence of shorter length of stay in hospital for patients sent to virtual wards compared to hospital inpatients. Virtual ward leads to a reduction in length of stay from 5.96 days to 2.89 days (a reduction of 3.07 days) on average, one of the shortest in the current literature as described above. The reduction in length of stay is achieved without an increase in the mortality of the virtual ward patients, although higher readmission rates and lower survival for virtual ward patients readmitted to hospital was found. Patients with no heart failure or CKD had shorter length of stay compared to the rest of the cohort.

The reduction in length of stay is an average 3.07 days for patients cared for by the virtual ward in 2022. Considering this cost was based on 366 virtual wards patients, this equates to 1123.62 hospital bed days freed in a year (3.07 x 366). This means that for a hospital with 100% occupancy (therefore providing 365 hospital bed days per bed) and 40 virtual wards beds at the capacity found in this study (24%), the virtual ward effect is an increase in the hospital capacity of 3.08 hospital beds per day (1123.62/365). The virtual ward saving of 3.08 hospital beds are around 1/9 of a whole typical 28 bedded ward. Therefore, 360 virtual ward beds are necessary to replace 28 hospital beds. The provision of 360 virtual ward would be an extensive undertaking and assumes that within the Trust there are patients who could appropriately be admitted to the 360 virtual ward beds. However, increasing the capacity to use the 40 virtual ward beds and reducing the time from hospital admission to virtual ward admission, can reduce the costs of a freed day hospital bed. However, given the current virtual ward organisation, it is therefore unlikely that the virtual ward could replace a whole hospital ward using the virtual ward model as it was studied in this paper. Nor is it more cost effective than traditional inpatient care. The original funding award agreed for the virtual ward was £4.4 million and would allow for a virtual ward of 160 virtual beds. That funding on the basis of this study would provide the replacement for approximately 12.5 hospital beds. But if the throughput of the virtual ward were to be doubled, then the virtual ward could replace almost a whole inpatient ward. The virtual ward would have 160 beds and would be staffed by 50 nurses and 28 additional HCA / ancillary staff – significantly greater than the requirement of the equivalent inpatient ward (35 WTE nurses and 12 WTE HCA/ancillary).

If the total cost could have covered the potential 14600 bed days from 40 virtual ward beds, than the cost for a virtual bed per day was around £72, a cost comparable to others reported in literature (i.e. €88 for virtual wards designed for severe chronic respiratory diseases [21]). However, the actual cost was of £299 for each of the 3508 bed days. In total, the cost for a patient in-hospital was £3194 on average, and that for a virtual ward patient (including the time spent in hospital) was £4336 on average. This is in contrast with some studies that have reported savings up to €8000 from virtual wards, although the studies were of not high quality [3 7], and not comparable to current UK NHS provision.

Whilst the virtual ward is shown to be an effective way to reduce hospital stay, it requires more carers and more cost than a traditional hospital stay. To be cost-effective the virtual ward would need to double its throughput without altering the standard of care or type of patients cared for. At that level, the service would be cost effective, but would still not provide a saving on traditional inpatient care. Virtual ward care has more readmissions and looks after patients with fewer comorbidities. To provide a saving on traditional care it would need to aspire to tripling throughput. Without further efficiency in the virtual ward provision [23],

[b] The Times 'NHS virtual wards available for 10,000 patients by September' accessed 01/08/2023 at https://www.thetimes.co.uk/article/nhs-virtual-wards-patients-amanda-pritchard-7v0xskh2k

For peer review only - http://bmjopen.bmj.com/site/about/guidelines.xhtml

it remains a clinically effective way to care for patients but not a cost effective or efficient way to care for patients.

Acknowledgments

We thank the WWL financial and healthcare teams for supporting this work, and all the WWL patients, which data contributed to the findings of this work.

Contributors

All authors conceived and designed the study. AU and MF collected and screened the data. AJ and LS analysed and interpreted the data. AJ and LS drafted the first version of the manuscript. All authors critically revised the manuscript for important intellectual content. MF and LS supervised the study. AU and MF had full access to all the data in the study and take responsibility for the integrity of the data and the accuracy of the data analysis. MF is the guarantor. The corresponding author attests that all listed authors meet authorship criteria and that no others meeting the criteria have been omitted.

Funding

No funding was available for this research.

Competing interests

All authors have completed the ICMJE uniform disclosure form at www.icmje.org/disclosure-of-interest/ and declare: LS's institution received consultancies fees from WWL for the submitted work. MF is the Associate Medical Director at Wrightington, Wigan and Leigh Teaching Hospitals. AU and AJ declare no conflict of interest.

Ethical approval

Ethics application for use of anonymised secondary data has been approved by the Faculty of Health and Medicine Ethics Committee on the 13th of June 2023 (FHM REC Reference: FHM-2023-3699-DataOnly-3. Title: Evaluation of virtual wards in a medium size hospital in UK).

Data sharing

Data are available on reasonable request. All data were anonymised and data sharing followed the Trust Governance protocols and procedures. Identifiable patient-level data from this project are not available to the public. Data are available on reasonable request from martin.farrier@wwl.nhs.uk.

The lead author (LS) affirms that the manuscript is an honest, accurate, and transparent account of the study reported; no important aspects of the study have been omitted.

For peer review only - http://bmjopen.bmj.com/site/about/guidelines.xhtml

Dissemination to participants and related patient and public communities

Dissemination of this study to patients, clinicians, researchers, and the public will be done through Lancaster University website and social media; WWL website and social media; through the researchers' private social media accounts including LinkedIn and Twitter; and through a dedicated press release with a plain language summary, including direct engagements and interview with the media, locally and globally. We will share the study's information and conclusions with NHS boards, clinicians, patients and parliamentary groups. A public lecture will be organised in Wigan by Lancaster University.

Provenance and peer review

Not commissioned; externally peer reviewed.

REFERENCES

1. Best J. The virtual wards aiming to ease hospital pressures. *Bmj-Brit Med J* 2022;378 doi: 10.1136/bmj.o1603
2. Alboksmaty A, Beaney T, Elkin S, et al. Effectiveness and safety of pulse oximetry in remote patient monitoring of patients with COVID-19: a systematic review. *Lancet Digit Health* 2022;4(4)
3. Norman G, Bennett P, Vardy ERLC. Virtual wards: a rapid evidence synthesis and implications for the care of older people. *Age Ageing* 2023;52(1) doi: 10.1093/ageing/afac319
4. Eines TF, Storm M, Gronvik CKU. Interprofessional collaboration in a community virtual ward: A focus group study. *Scand J Caring Sci* 2023 doi: 10.1111/scs.13152
5. Creavin ST, Garg M, Hay AD. Impact of remote vital sign monitoring on health outcomes in acute respiratory infection and exacerbation of chronic respiratory conditions: systematic review and meta-analysis. *Erj Open Res* 2023;9(2) doi: 10.1183/23120541.00393-2022
6. Kanyimo E. Using virtual wards and long-term conditions management network to improve practice and performance. *Bmj Open Qual* 2022;11(4) doi: 10.1136/bmjoq-2022-001952
7. Chauhan U, McAlister FA. Comparison of Mortality and Hospital Readmissions Among Patients Receiving Virtual Ward Transitional Care vs Usual Postdischarge Care A Systematic Review and Meta-analysis. *Jama Netw Open* 2022;5(6) doi: 10.1001/jamanetworkopen.2022.19113
8. Lasserson D, Cooksley T. Virtual wards: urgent care policy must follow the evidence. *Bmj-Brit Med J* 2023;380 doi: 10.1136/bmj.p343
9. Dhalla IA, O'Brien T, Morra D, et al. Effect of a Postdischarge Virtual Ward on Readmission or Death for High-Risk Patients A Randomized Clinical Trial. *Jama-J Am Med Assoc* 2014;312(13):1305-12. doi: 10.1001/jama.2014.11492
10. Rue H, Martino S, Chopin N. Approximate Bayesian inference for latent Gaussian models by using integrated nested Laplace approximations. *Journal of the Royal Statistical Society: Series B (Statistical Methodology)* 2009;71(2):319-92. doi: https://doi.org/10.1111/j.1467-9868.2008.00700.x
11. Moody S, Lamb T, Jackson E, et al. Assessment and management of secondary bacterial infections complicating Mpox (Monkeypox) using a telemedicine service. A prospective cohort study. *Int J Std Aids* 2023;34(7):434-38. doi: 10.1177/09564624231162760
12. Ward TJC, Mavilakandy A, Danns J, et al. Outcomes from a virtual ward delivering oxygen at home for patients recovering from COVID-19: a real world observational study. *Clin Med* 2022;22(3):197-202. doi: 10.7861/clinmed.2021-0512

For peer review only - http://bmjopen.bmj.com/site/about/guidelines.xhtml

13. Uminski K, Komenda P, Whitlock R, et al. Effect of post-discharge virtual wards on improving outcomes in heart failure and non heart failure populations: A systematic review and meta-analysis. *Plos One* 2018;13(4) doi: 10.1371/journal.pone.0196114
14. Majoor K, Vorselaars ADM. Home monitoring of coronavirus disease 2019 patients in different phases of disease. *Curr Opin Pulm Med* 2023;29(4):293-301. doi: 10.1097/Mcp.0000000000000964
15. Mackay I, France M, McAuley D, et al. COVID-19 (Omicron strain) hospital admissions from a virtual ward - who required further care? *Influenza Other Resp* 2023;17(3) doi: 10.1111/irv.13108
16. Ko SQ, Chua CMS, Koh SH, et al. Experiences of patients and their caregivers in a Virtual Ward in Singapore: A descriptive qualitative study. *Int J Med Inform* 2023;177 doi: 10.1016/j.ijmedinf.2023.105111
17. van Ramshorst J, Duffels M, de Boer SPM, et al. Connected care for endocarditis and heart failure patients: a hospital-at-home programme. *Neth Heart J* 2022;30(6):319-27. doi: 10.1007/s12471-021-01614-2
18. Kirkcaldy A, Jack BA, Cope LC. Health care professionals' perceptions of a community based 'virtual ward' medicines management service: A qualitative study. *Res Soc Admin Pharm* 2018;14(1):69-75. doi: 10.1016/j.sapharm.2017.02.001
19. Tan MWJ, Arciga MGA, Arba'in JB, et al. Outcomes of a "hospital at home" programme for the supervised home recovery of COVID-19 patients in Singapore. *Proc Singap Healthc* 2023;32 doi: 10.1177/20101058231152049
20. O'malley EJ, Hansjee S, Abdel-Hadi B, et al. A Covid -19 Virtual Ward Model: A Preliminary Retrospective Clinical Evaluation From a UK District General Hospital. *J Prim Care Communit* 2022;13 doi: 10.1177/21501319211066667
21. Cushen B, Madden A, Long D, et al. Integrating hospital and community care: using a community virtual ward model to deliver combined specialist and generalist care to patients with severe chronic respiratory disease in their homes. *Irish J Med Sci* 2022;191(2):615-21. doi: 10.1007/s11845-021-02633-z
22. Lewis C, O'Caoimh R, Patton D, et al. Utilisation of a Suite of Screening Tools to Determine Adverse Healthcare Outcomes in an Older Frail Population Admitted to a Community Virtual Ward. *Int J Env Res Pub He* 2021;18(11) doi: 10.3390/ijerph18115601
23. van der Lande ACMH, Treskes RW, Beeres SLMA, et al. Continuum of Care: Positioning of the Virtual Hospital. *Front Cardiovasc Med* 2022;8 doi: 10.3389/fcvm.2021.779075
24. Guest JF, Keating T, Gould D, et al. Modelling the annual NHS costs and outcomes attributable to healthcare-associated infections in England. *BMJ Open* 2020;10(1):e033367. doi: 10.1136/bmjopen-2019-033367 [published Online First: 2020/01/25]

For peer review only

For peer review only - http://bmjopen.bmj.com/site/about/guidelines.xhtml

Length of stay and economical sustainability of virtual ward care in a medium sized hospital of the UK. A retrospective case-control study

Abdollah Jalilian[1,*], Luigi Sedda[1,*,$], Alison Unsworth[2] and Martin Farrier[2,#]

For peer review only

Supplementary information

Supplemental Table S1. Comparison of matching health indicators between all 366 patients admitted to virtual wards and the 318 virtual ward patients who were matched with patients admitted exclusively to the hospital. Based on t-tests for equality of means and chi-square tests for equality of proportions, no significant differences were found between the two groups at the significance level of 5%.

	Matched virtual ward patients (318)	All virtual ward patients (318+48)
Average CFS score	3.97	3.99
Average age	67.7	67.97
Average length of stay in virtual wards (from virtual wards onboarding to virtual wards offboarding)	9.79 days	9.64 days
Total number of virtual ward days	3114	3508
Number (percentage) of female patients	174 (54.7%)	203 (55.5%)
Number (percentage) of patients with diabetes	47 (14.8%)	54 (14.6%)
Number (percentage) of patients with hypertension	103 (32.4%)	125 (34.2%)
Number (percentage) of patients with chronic heart disease	36 (11.3%)	45 (12.3%)
Number (percentage) of patients with chronic kidney disease	2 (0.6%)	7 (1.9%)
Number (percentage) of patients with chronic obstructive pulmonary disease	81 (25.5%)	90 (24.6%)
Number (percentage) of patients with asthma	8 (2.5%)	14 (3.8%)

For peer review only - http://bmjopen.bmj.com/site/about/guidelines.xhtml

Supplemental Table S2. Costs for the provision of virtual ward for the calendar year of 2022.

Item	Whole Time Equivalent	Cost £
Nurses (band 5 and above)	12.5	520,032
Healthcare Assistant / Ancillary / Admin (Band 4 and below)	6.8	189,538
Pharmacists	2	122,239
Consultants	0.5	46,709
Corporate non clinical	1.45	148,165
Hardware		5,874
Travel		935
Medical Equipment		13,529
Additional Costs		4,129
Total		1,051,150

For peer review only

STROBE checklist compliance

Strobe Statement

Title and abstract

1a and b Compliant

Introduction

2 and 3 Compliant

Methods

4 and 5 Compliant

6 As far as is possible this is explained in the text. Matching criteria are included.

7 Compliant

8 Compliant. The sources of data are the same for each group.

9 Compliant. This is addressed in the text.

10 Compliant. This is addressed in the text. The study size was "all patients"

11Compliant. This is addressed in the text.

12 Compliant. This is addressed in the text

Participants

13 Compliant. There are 2 stages, the study period (2022) and a check on whether the patient is alive at 30th April 2021. All patients are considered at both stages. There is no additional value in a flow diagram.

14 Compliant. This is addressed in the text.

15 Compliant.

16 Compliant.

17 Compliant.

Discussion

18 Compliant.

19 Compliant.

20 Compliant.

For peer review only

21 Compliant.

Other information

22 DOI completed for each of the authors. No funding has been received for this work.

For peer review only

BMJ Open

Length of stay and economical sustainability of virtual ward care in a medium sized hospital of the UK. A retrospective longitudinal study

Journal:	*BMJ Open*
Manuscript ID	bmjopen-2023-081378.R1
Article Type:	Original research
Date Submitted by the Author:	22-Dec-2023
Complete List of Authors:	Jalilian, Abdollah; Lancaster University<br>Sedda , Luigi ; Lancaster University<br>Unsworth, Alison; Wrightington Wigan and Leigh NHS Foundation Trust<br>Farrier, Martin; Wrightington Wigan and Leigh NHS Foundation Trust, Paediatrics
**Primary Subject Heading**:	Health services research
Secondary Subject Heading:	Health policy
Keywords:	Telemedicine < BIOTECHNOLOGY & BIOINFORMATICS, EPIDEMIOLOGIC STUDIES, HEALTH ECONOMICS, Health Services

SCHOLARONE™
Manuscripts

I, the Submitting Author has the right to grant and does grant on behalf of all authors of the Work (as defined in the below author licence), an exclusive licence and/or a non-exclusive licence for contributions from authors who are: i) UK Crown employees; ii) where BMJ has agreed a CC-BY licence shall apply, and/or iii) in accordance with the terms applicable for US Federal Government officers or employees acting as part of their official duties; on a worldwide, perpetual, irrevocable, royalty-free basis to BMJ Publishing Group Ltd ("BMJ") its licensees and where the relevant Journal is co-owned by BMJ to the co-owners of the Journal, to publish the Work in this journal and any other BMJ products and to exploit all rights, as set out in our licence.

The Submitting Author accepts and understands that any supply made under these terms is made by BMJ to the Submitting Author unless you are acting as an employee on behalf of your employer or a postgraduate student of an affiliated institution which is paying any applicable article publishing charge ("APC") for Open Access articles. Where the Submitting Author wishes to make the Work available on an Open Access basis (and intends to pay the relevant APC), the terms of reuse of such Open Access shall be governed by a Creative Commons licence – details of these licences and which Creative Commons licence will apply to this Work are set out in our licence referred to above.

Other than as permitted in any relevant BMJ Author's Self Archiving Policies, I confirm this Work has not been accepted for publication elsewhere, is not being considered for publication elsewhere and does not duplicate material already published. I confirm all authors consent to publication of this Work and authorise the granting of this licence.

For peer review only

Length of stay and economical sustainability of virtual ward care in a medium sized hospital of the UK. A retrospective longitudinal study

Abdollah Jalilian[1,*], Luigi Sedda[1,*,$], Alison Unsworth[2] and Martin Farrier[2,#]

[1]Lancaster Ecology and Epidemiology Group, Lancaster Medical School, Lancaster University, Lancaster, UK

[2]Wrightington Wigan and Leigh Teaching Hospitals NHS Foundation Trust, Wigan, UK

[*]These authors contributed equally to the work.

[$]Corresponding author: l.sedda@lancaster.ac.uk

[#]Senior author

Abstract

Objective

To evaluate length of stay difference and its economic implications between hospital patients and virtual ward patients.

Design

Retrospective longitudinal study.

Setting

Wrightington, Wigan and Leigh Teaching Hospitals National Health Service (NHS) Foundation Trust (WWL), a medium-sized NHS Trust in north-west of England.

Participants

Virtual ward patients (n=318) were matched 1:1 to 1:4, depending on matching characteristics, to all hospital patients (n= 350). All patients were admitted to hospital during the calendar year of 2022.

Outcome measures

The primary outcome is the length of stay as defined from the date of hospital admission to the date of discharge/death (hospital patients) and from the date of hospital admission to the date of admission in virtual ward (virtual ward patients). The secondary outcome is the cost of a hospital bed day and the equivalent value of virtual ward saving in hospital bed days. Additional measures were 6 months readmission rates and survival rates at the follow up date of 30th April 2023.

For peer review only - http://bmjopen.bmj.com/site/about/guidelines.xhtml

Risk factors

Age, sex, comorbidities and clinical frailty score were used to evaluate the importance and effect of these factors on the main and secondary outcome.

Methods

Statistical analyses included logistic and binomial mixed models for length of stay in hospital and readmission rate outcomes, and Cox proportional hazard model for the survival of the patients.

Results

The virtual ward patients had a shorter stay in hospital before being admitted to the virtual ward (2.89 days, 95% CI 2.1-3.9 days). Chronic kidney disease and frailty were associated to longer length of stay in hospital (58% 95% CI 22%-100% compared to patients without CKD and 14% 95%CI 8%-21% compared to patients with one unit lower CFS). The frailty score was also associated to higher rate of readmission within 6 months and lower survival. Being admitted to virtual ward slightly improved survival, although when readmitted the survival deteriorated rapidly. The cost of a 24 hour period in a general hospital bed is £536. The cost of a day hospital saved by virtual ward was £935.

Conclusion

The use of a 40 virtual bedded ward was clinically effective in terms of survival for patients not needing readmission and allowed for the freeing of 3 hospital beds per day. However, the cost for each day freed from hospital stay was three quarters larger than the one for a single day hospital bed. This raises concerns on the deployment of large scale virtual wards without the existence of policies and plans for their cost-effective management.

For peer review only

Strengths and limitations

1. The largest virtual wards study, in terms of virtual wards patients, and the first in UK assessing their cost.
2. The information about inclusion and exclusion criteria used to send patients to virtual wards was subjective and therefore subject to selection bias.
3. Exact costs for hospital patients were not available for this research, causing some uncertainty on the true cost difference between hospital and virtual ward patients.
4. Given the sample size, only few individual factors were used for matching, therefore not all potential confounders could have been considered.

For peer review only

For peer review only - http://bmjopen.bmj.com/site/about/guidelines.xhtml

Introduction

Virtual wards offer patients the opportunity to receive health care in the comfort and safety of their own homes, including care homes (1). Pioneered in the UK, virtual wards became a popular scheme during COVID-19 (2) and since then it has been replicated in other 'thematic virtual wards' (i.e. respiratory, heart failure and chronic obstructive pulmonary disease to name few). Virtual wards are the response to the chronic and unsustainable high demand for secondary care beds (3). By providing an alternative to continued admission, virtual ward may help bridge the gap between demand and capacity for hospital beds especially in the context of aging population (3, 4), although its successes are still debated (see for example Creavin and colleagues' review on randomised controlled trials of virtual wards for acute respiratory infection(5)). A plan to enrol virtual wards in most of the Integrated Care Systems in UK has been launched in 2020 with the aim to make available hundreds of thousands of virtual beds (at a cost of hundreds of million pounds) in the next years in order to provide them as an option for every patient (6).

Virtual Wards must be cost effective if they are to replace traditional inpatient care, the costs must be comparable or lower than the costs of hospital stay to be economically sustainable in the medium to long terms. To establish whether large scale implementation of virtual wards could alter the provision of healthcare, it is necessary therefore to address workforce issues and reduce the cost of modern healthcare.

The main objective of this research is to compare the length of stay in a medium sized UK hospital for hospital and virtual wards admitted patients. We define the length of stay as the duration, in days, between the date of admission to the hospital and the date of discharge from the hospital for hospital patients, and from date of admission to the hospital and the date of admission to virtual ward for virtual ward patients. By analysing the length of stay, we aim to assess the effectiveness of virtual wards in reducing the duration of hospitalization and therefore increasing the number of beds available for hospital patients. This is what is also defined as 'step-down' model (3).

Beyond potential reductions in the length of a patient stay in hospital, we aim to compare the survival outcomes of patients in both virtual wards and hospital settings. Additionally, we compare the readmission rates to the hospital within six months of discharge. Survival and readmission are two indicators of the clinical effectiveness of the virtual ward to satisfy the healthcare needs of the patients (7) .

By considering all these aspects—length of stay, survival, readmission rates and costs, it will be possible to assess the impact of virtual wards on patient care, and its effectiveness in comparison to traditional inpatient care. The findings from this study respond to recent requests on evidence-based informed virtual wards provision (8), and will contribute to a deeper understanding of the potential benefits and implications of virtual wards as an alternative care model.

For peer review only - http://bmjopen.bmj.com/site/about/guidelines.xhtml

Material and methods

Virtual Ward

The Virtual Ward at Wrightington, Wigan, and Leigh Teaching Hospitals National Health Service Foundation Trust (WWL) initially originated as an oxygen service to facilitate the follow-up of COVID-19 patients. As the number of COVID-19 cases requiring follow-up decreased, the service underwent repurposing. The administration of virtual ward patient management utilised a software platform provided by Current Health, with monitoring equipment established by WWL medical electronics team to generate automated data through the Current Health web-based system. By the onset of 2022, the virtual ward had evolved into a stable service, providing a viable alternative to continuous hospital admissions.

A dedicated team of core nurses maintained daily communication with patients through iPad/FaceTime. Patient data is systematically scrutinised through the analysis of automated observations within a cloud-based software framework. In instances where concerns arise about a patient's condition, virtual ward nurses coordinate a review with the responsible Consultant. In-person visits for tasks such as intravenous antibiotic administration, blood sample collection, or wound management were organised by the nurses. Continuous monitoring is conducted to track the patient's progress or detect signs of deterioration. In cases of deterioration, arrangements for readmission can be made, with transportation facilitated to the Emergency Department or Same Day Emergency Care through ambulance services if necessary.

The selection of patients for the virtual ward is a collaborative effort between medical and nursing teams on the ward. The virtual ward team conducts assessments to determine suitability, and upon confirmation, a technical setup visit is arranged at the patient's residence to verify self-sufficiency, assistance from a partner or family, or an existing care support system. Patients relocated to care homes are excluded from virtual ward consideration.

Discharge from the virtual ward is contingent upon assessments conducted by the virtual ward team and only when monitoring is deemed unnecessary for the patient.

Data

The study follows a retrospective longitudinal design where virtual ward patients undergo a partial discharge process from the hospital and continue to receive medical attention and support within their homes until they complete their treatment and are then fully discharged; and hospital patients receive care within the hospital until they are fit for full discharge.

Virtual ward patients are matched manually with patients admitted exclusively to the hospital, resulting in 350 virtual ward-hospital pairs. Patient Administration System data was used to pull a list of age matched patients who were admitted during the same study year. Further data from coding was then used to provide full matching for virtual ward patients. These patient pairs comprised 350 unique hospital patients and 318 virtual ward patients. Therefore 291 are matched 1virtual ward to 1hostpital patient; 23 are 1:2; 3 are 1:3 and finally 1 is 1:4. The study group comprises all the patients admitted to Wrightington, Wigan and Leigh Teaching Hospitals NHS Foundation (WWL) during the calendar year 2022 (1st of January 2022 to the 31st of December 2022) and their progress was followed up on the 30th of April 2023.

Matching characteristics were sex, age, primary diagnosis description and Clinical Frailty Score (CFS, 1 representing a very fit patient to 9 indicating a terminally ill patient). CFS is measured at triage by the care

For peer review only - http://bmjopen.bmj.com/site/about/guidelines.xhtml

professional seeing the patient (it may also be reassessed at a later point in the admission by other healthcare professionals if further information becomes available). Attribution of one of the CFS score to a patient was carried with the support of the Clinical Frailty Scale App (9).

In particular, all matched patients have the same gender and the same primary diagnosis description. 97% of the matched patients possess the same CFS score, while the remaining 3% have a CFS scores within a range of ±2 points. Finally, 95% of the matched patients share the same age or have an age difference within ±6 years, with the remaining 5% having an age gap of less than 12 years. Matching was exact where possible but for a minority of patients an exact match was not possible in the dataset for the study year. In that case the closest possible match was achieved leading to the ranges in CFS and age.

Additional unmatched information was provided for each patient: comorbidities, readmission information, date of admission and discharge to and from hospital, date of admission and discharge to and from virtual ward and date of death when applicable up to 30th April 2023. ICD-10 based coding data was employed to assign comorbidities to each patient. Each patient's episodes of care were meticulously coded at the point of discharge, offering comprehensive insights into both the reason for admission and the documented co-morbidities. These co-morbidities were then cross-referenced with the records obtained at the time of admission to the virtual ward. In instances where disparities were identified between the recorded co-morbidities at admission and those during the episodes of care, the electronic notes of each patient were meticulously examined to establish the accurate set of co-morbidities. The electronic notes served as the reference in resolving any discrepancies, ensuring the utmost precision in our data interpretation. Due to the study design and rigorous data entry checks, there is no missing data.

48 patients admitted to virtual wards did not have suitable matches among the patients admitted exclusively to the hospital during the study year. Consequently, these 48 patients were excluded from the analyses.

To estimate the cost-effectiveness of virtual ward admission compared to exclusively hospital ward admission the costs of facilities and staffing were included in the analysis. Financial data for the cost of the virtual ward was maintained separate from other Trust Finances so that the cost of the virtual ward could be fully understood. Financial data were provided by the WWL Trust Finance Team.

Patient and public involvement

Owing to use of retrospective data sources, no members of the public or patients were formally involved in the study's design, analysis, or manuscript writing, due to the limited size of the research group and the absence of funding. The communication plan includes executive summaries circulated at the NHS Trust, press releases, public lectures, and social media posts to reach the most diverse audience.

For peer review only

Methods

Study objectives and respective methods are summarized in Table 1. Because some virtual ward patients have been matched with more than one hospital patient, a random effect representing recurring virtual ward patients is included in the models.

Table 1. Statistical analyses employed for the four study outcomes.

	Primary outcome	Secondary outcome	Additional outcomes	
	Length of stay (in days)	Bed cost	Being readmitted to the hospital within 6 months after discharge (10)	Survival at the follow up date of 30/4/2023
**Question**	Which are the factors influencing the length of stay in the hospital?	What is the daily cost of a bed in hospital and a bed in virtual ward?	What are the factors associated to readmission of patients within 6 months after discharge?	What are the factors contributing to the survival of patients?
**Statistical Model**	Generalized mixed model with generalized Poisson response	Descriptive approach: direct collation of cost for Virtual Ward compared with NHS approved methodology for non-elective inpatient bed cost at the same organisation.	Logistic regression model	Cox proportional hazard model

Models were run within a Bayesian framework with assigned non-informative prior distributions to all parameters. The computations were performed by using the R-INLA package (11) to allow to incorporate uncertainty in the data and estimate the posterior distributions of the model parameters using the INLA methodology (11).

Virtual ward cost is calculated from accrued expenditure. This was kept as a separate spreadsheet so that exact costs were known. The virtual ward utilizes 12.5 Whole Time Equivalent (WTE) nurses and 0.5 WTE Consultants with the assistance of 2 WTE pharmacists and 6.8 WTE ancillary staff members. NICE recommendations(12) provide a basis for the calculation of standard ward staffing for an idealized inpatient ward. Using their recommendations, a typical 28 bedded hospital ward would require 35 WTE nurses to staff with 12 WTE ancillary staff members. Medical staffing for a traditional 28 bedded ward would depend on the clinical work but would require a ward round each day and the involvement of a Consultant and Junior medical staff.

Cost estimation for hospital bed day uses NHS agreed methodology. It represents the average cost of non-elective care provision in wards on the Acute Hospital site. It is calculated by the Finance Department at the organisation and is in line with the costs submitted by the organisation to NHS England.

For peer review only - http://bmjopen.bmj.com/site/about/guidelines.xhtml

Patients who are cared for in the virtual ward are by definition in the second half of their inpatient hospital stay. For hospital patients, costs are higher for the first day of a patient admission to hospital than they are for subsequent days because patients are likely to need more care, tests and intervention in the first part of their stay. At the moment an estimate of cost for the second half of a patients stay in hospital is not available, only the average cost for all days spent in hospital has been provided. As a consequence, the estimate of hospital stay is a high estimate, and the virtual ward cost is an exact cost.

Results

Summative and descriptive analysis

The primary diagnosis description for the majority (64.3%) of the matched patients corresponds to the conditions pneumonia, chronic obstructive pulmonary disease (COPD), COVID-19, atrial fibrillation, or heart failure (HF). Overall, patients admitted to virtual wards exhibit a shorter hospital stay in comparison to their matched counterparts in the hospital (p-value < 0.001) (Table 2). Based on a 95% confidence interval, it is estimated that virtual ward patients experience a reduction in hospital stay ranging from 2.1 to 3.9 days compared to their corresponding matches in the hospital.

Table 2. Baseline demographics of virtual ward and hospital patients. Number of stars resulting from the p-value obtained from a paired t-test: * if 0.01 ≤ p-value < 0.05, ** if 0.001 ≤ p-value < 0.01 and *** if p-value < 0.001. No stars indicate a p-value above 0.05. For comorbidities, only those above 2% are shown in the table.

Baseline demographics	Patients admitted only to the hospital (n = 350)	Patients admitted to virtual wards (n = 318)	p-value
Percentage of female patients	54.6%	54.7%	
Median age (years)	72	71	
Median CFS score	4	4	
Number (percentage) of patients with Pneumonia as their primary diagnosis description	70 (20%)	61 (19.2%)	
Number (percentage) of patients with Chronic obstructive pulmonary disease as their primary diagnosis description	63 (18%)	55 (17.3%)	
Number (percentage) of patients with COVID as their primary diagnosis description	38 (10.9%)	33 (10.4%)	
Number (percentage) of patients with Atrial Fibrillation as their primary diagnosis description	30 (8.6%)	27 (8.5%)	
Number (percentage) of patients with Heart Failure as their primary diagnosis description	24 (6.9%)	22 (6.9%)	
Number (percentage) of patients with Hypertension as their primary diagnosis description	15 (4.3%)	14 (4.4%)	
Number (percentage) of patients with Cellulitis as their primary diagnosis description	11 (3.1%)	11 (3.4%)	
Number (percentage) of patients with Chest infection as their primary diagnosis description	10 (2.9%)	10 (3.1%)	
Average length of stay in the hospital (from hospital admission to hospital discharge or to virtual ward)	5.96 days	2.89 days	***

For peer review only - http://bmjopen.bmj.com/site/about/guidelines.xhtml

Average length of stay in virtual wards (from virtual wards onboarding to virtual wards offboarding)	---	9.79 days	NA
Total number of hospital days used by patients	2086	920	
Total number of virtual wards days used by patients	---	3114	
Average length of care in both hospital and virtual wards	5.96 days	12.96 days	***
Number (percentage) of patients with diabetes	86 (24.6%)	47 (14.8%)	**
Number (percentage) of patients with hypertension	159 (45.4%)	103 (32.4%)	***
Number (percentage) of patients with chronic heart disease	123 (35.1%)	36 (11.3%)	***
Number (percentage) of patients with chronic kidney disease	51 (14.6%)	2 (0.6%)	***
Number (percentage) of patients with chronic obstructive pulmonary disease	128 (36.6%)	81 (25.5%)	**
Number (percentage) of patients with asthma	39 (11.1%)	8 (2.5%)	***
Number (percentage) of patients with learning disability	5 (1.4%)	1 (0.3%)	
Number (percentage) of patients being readmitted to the hospital within 6 months after discharge	108 (30.9%)	128 (40.3%)	*
Number (percentage) of patients being alive on the follow up date 30/4/2023	275 (72.4%)	275 (86.5%)	*

In general, patients admitted to virtual wards have lower prevalence rates of asthma, HF, chronic kidney disease (CKD), COPD, diabetes, hypertension, and learning disability compared to the patients staying in hospital. Moreover, a slightly higher percentage of patients admitted to virtual wards were alive on the follow-up date in comparison to patients admitted exclusively to the hospital (although bordering statistical significance). Finally, readmission rate within six months after discharge is higher for patients admitted to virtual wards than hospital patients. Only one patient was re-admitted to hospital during the virtual ward clinic.

Virtual wards patients used to match hospital patients were representative of the total virtual ward patient cohort (Supplementary Table S1).

Length of stay in hospital, readmission and survival models.

The results of the logistic regression model to the binary variable of being readmitted within 6 months of discharge (summarised in Table 3) indicates that several factors are significantly influencing the likelihood of readmission within 6 months of discharge. Older age and higher CFS score, and having COPD, are associated with increased chances of readmission. Specifically, for every one-unit increase in age, while keeping all other factors at their average level, there is a 3% increase in the odds of readmission. Similarly, for each unit increase in the CFS score, while holding all other factors at their average level, the odds of readmission increases by 20%. Having a diagnosis of COPD leads to a 70% higher odds of readmission. Notably, being admitted to virtual wards is the most substantial risk factor for readmission within the next 6 months. Virtual ward patients face a significant 120% increased odds of readmission compared to their counterparts in the hospital.

Table 3. Odds ratios and their corresponding 95% credible intervals for factors affecting the probability of being readmitted within 6 months of attendance (in bold the statistically significant factors at a significance level of 5%).

	odds ratio	95% credible interval	
Intercept	0.033	0.01	0.083

For peer review only

Gender Male	0.756	0.531	1.044
**Age**	**1.027**	**1.011**	**1.043**
**CFS score**	**1.176**	**1.035**	**1.329**
Diabetes	1.441	0.924	2.146
Hypertension	0.732	0.508	1.022
Heart failure	0.955	0.61	1.424
Chronic kidney disease	1.408	0.7	2.54
**Chronic obstructive pulmonary disease**	**1.673**	**1.148**	**2.356**
Asthma	0.949	0.428	1.833
Learning disability	9.104	0.937	36.551
Length of stay	1.023	0.997	1.048
**Admitted to virtual wards**	**2.226**	**1.506**	**3.172**

The results from the Cox proportional hazard model applied to the survival from admission to either death or being alive at the follow up date are shown in Table 4. The proportional hazards assumption for the fitted Cox model was validated (p-value = 0.11), indicating that the hazards were proportional over time. Based on these results the following conclusions can be made:

1. Patients admitted to virtual ward had a significant lower mortality (63%) compared to those admitted to hospital.
2. Patients with heart failure show a 75% higher risk of death compared to those without this condition.
3. For each unit increase in the CFS score, while holding all other factors at their average level, the risk of death increases by approximately 35%.
4. There is a 4% increase in the risk of death for each additional day of hospital stay, while keeping all other factors at their average level.
5. Patients who are readmitted within 6 months of discharge face a significant increase in the risk of death. Specifically, virtual wards patients readmitted in hospital experience a 220% higher risk of dying compared to the 175% of hospital patients readmitted to hospital when compared to non-readmission patients.

Table 4. Hazard ratios and their corresponding 95% credible intervals for factors affecting the death of patients from the date of admission (in bold the statistically significant factors at a significance level of 5%).

	hazard ratio	95% credible interval	
Intercept	0.001	0	0.004
Gender Male	1.03	0.703	1.457
Age	1.009	0.99	1.028
**CFS score**	**1.351**	**1.181**	**1.538**
Diabetes	0.966	0.605	1.465
Hypertension	0.915	0.615	1.311
**Hearth Failure**	**1.762**	**1.143**	**2.597**
Chronic kidney disease	1.047	0.556	1.8
Chronic obstructive pulmonary disease	1.376	0.937	1.951
Asthma	0.405	0.104	1.097

For peer review only - http://bmjopen.bmj.com/site/about/guidelines.xhtml

Learning disability	0.867	0.065	3.843
**Admitted to virtual ward**	**0.370**	**0.160**	**0.750**
**Length of stay**	**1.04**	**1.02**	**1.06**
**Readmission 6 months: hospital patient**	**2.763**	**1.688**	**4.272**
**Readmission 6 months: Admitted to virtual wards**	**3.179**	**2**	**4.805**

Table 5: Risks and their corresponding 95% credible intervals for factors associated to the length of stay in hospital of patients (in bold the statistically significant factors at a significance level of 5%).

	Exp(coefficients)	95% credible interval	
Intercept	4.116	2.566	6.29
Gender Male	0.938	0.796	1.099
Age	0.995	0.988	1.002
**CFS score**	**1.144**	**1.077**	**1.213**
Diabetes	1.103	0.91	1.323
Hypertension	1.035	0.874	1.217
Hearth Failure	1.134	0.918	1.383
**Chronic kidney disease**	**1.579**	**1.224**	**2.004**
Chronic obstructive pulmonary disease	1.077	0.872	1.315
Asthma	1.163	0.867	1.527
Learning disability	1.287	0.651	2.29
Readmission within 6 Months	1.084	0.918	1.271
**Admitting to virtual wards**	**0.563**	**0.47**	**0.669**

Finally, the length of stay is associated to several factors (Table 5):

1. Having CKD is associated with an average increase of around 60% in the length of stay.
2. For each unit increase in the CFS score, while holding all other factors at their average level, there is an average increase of around 15% in the length of stay.
3. Being admitted to virtual wards, as opposed to staying in the hospital, results in an average decrease of around 45% in the length of stay in hospital before admission to virtual ward.

Including an interaction term for each co-morbidity and hospital treatment (virtual ward or inpatient hospital), does not produce statistically significant coefficients for any of these terms.

Hospital and virtual ward costs

The costs for the virtual ward during the Calendar Year 2022 are shown in Supplementary Table S2. The total was around £1.051 million for 40 virtual ward beds. There were additional costs of £100,000 as part of the process of setting up the service, but these are non-recurrent costs and therefore not included in the final costings for the virtual ward.

The calculated reduction of in-patient hospital bed days for patients admitted to the virtual ward was 3.07 days for each of the patients admitted to the virtual ward (Table 2, 5.96 vs 2.89 days). There were 366 patients admitted to the virtual ward during the study period for which the costs of the virtual ward were £1.051

For peer review only

million. Using those figures, the cost of saving one in-patient hospital day was £935 (£1,051,150/(3.07 days x 366 patients)).

The average cost of a non-elective in-patient bed day within the organisation using standard NHS methodology was provided by the WWL Finance Department and amounted to £536, a cost coherent with other studies (13).

Comparison of costs for the virtual ward with the costs of a continued inpatient stay is therefore possible. The cost of reducing a patients hospital stay by one day is £935, when they are admitted to the virtual ward as a strategy for shortening their in-patient stay. If the patient had stayed in hospital rather than being admitted to the virtual ward, the equivalent in-patient hospital day cost would have been £536. Therefore, the cost of virtual ward care is approximately three quarters higher than that of traditional in-patient care. However, the £935 are calculated based on the WWL capacity to use the virtual beds, which was 24% of the potential 14,600 24 hour beds per year provided by 40 virtual wards beds (Supplementary Table S1, reference to 3,508 total days spent by virtual ward patients).

Discussion

This study has shown that virtual ward patients had a shorter length of stay in hospital, but more likely to be re-admitted in hospital, and when re-admitted have a lower chance to survive compared to non-readmitted patients and readmitted patients previously discharged from hospital. Virtual ward patients were affected by a lower number of comorbidities, although their frailty scores along with sex, age and primary diagnosis were matched with hospital patients. Whilst virtual wards are shown to effectively reduce the length in-patient stay, its cost associated to a freed day hospital bed is three quarters higher than that of in-patient hospital care.

Previous studies have focused on the development of virtual wards for specific conditions or group of patients. For the UK, one of the first countries that trialled virtual wards, literature is scarce and the size of virtual wards are smaller compared to the one presented here (3, 14, 15). In addition, this study is the first example of the assessment of virtual ward cost effectiveness in UK within a medium size hospital facility.

In terms of virtual ward clinical outcomes, current knowledge provides conflicting evidence (16) apart from randomised controlled trails enrolling patients with heart failure (7). Although a direct comparison of the present study with current literature over the clinical outcomes is not possible, some common patterns can be highlighted.

As in other studies, patients admitted to the virtual ward had lower rates of comorbidity (see for example the review for virtual ward COVID-19 by (17)). While recent literature reviews found lower rate of re-admission in COPD older patients (3) and COVID-19 patients (18) after hospital at home and virtual wards, the present study did not find any significant difference as also reported in (5). However, readmission rates for all patients (hospital and virtual wards) increased if a patient was from a virtual ward, older and with COPD. The higher rate of readmission is coherent with clinical expectations. The care provided by the virtual ward is, in part, a monitoring process, therefore clinical deterioration is often followed by readmission of the patient (14). For discharged hospital patients, readmission would need to be initiated by the patient. For those virtual ward patients not readmitted, the care is shown to be non-inferior, and other studies have indicated that care in virtual wards is preferred by patients and also by the healthcare staff (3, 19-21).

Survival was better in patients admitted to the virtual ward but decreases in patients from virtual wards when readmitted, and for those with hearth failure. A similar result was found in other studies (3, 10).

For peer review only - http://bmjopen.bmj.com/site/about/guidelines.xhtml

Although virtual ward patients were matched by sex, age, primary diagnosis description and CFS, they showed a lower frequency of co-morbidities than in the hospital patients which may have contributed to larger survival in their first admission compared to hospital patients. It is likely that, despite the careful matching of patients, there is a selection bias towards fitter patients when patients are being selected for consideration of admission to the Virtual Ward. Greater independence and ability to communicate with Virtual Ward staff by videophone make patients better candidates for admission to the Virtual Ward.

Our analysis found a length of stay in hospital prior to virtual ward admission to be shorter than the one in other studies, although many of them were based on COVID-19 virtual wards or other disease-specific virtual wards (5, 20, 22-24). A common result, however, is a shorter stay in hospital for virtual wards patients than hospital patients. The present study highlighted that apart from CKD, the reduction in length of stay is not related to any other primary diagnosis, although dependent on the assessed CFS. Length of stay increases in patients with higher CFS and/or with CKD.

It is important to note that CFS is detrimental for all the clinical outcomes considered in this analysis: length of stay, readmission and survival. Another study, although on an elderly cohort, shown similar association between CFS and mortality (25). These findings may suggest that patients with lower CFS are those that may benefit more from virtual wards in terms of cost-effectiveness and clinical outcome. Clinical experience indicates that virtual ward care requires a patient to have some self-caring ability. The more compromised that is, the less likely a virtual ward is able to provide appropriate levels of care.

The exclusion of co-morbidity indexes, such as the Charlson(26) or Elixhauser(27), in our study was driven by two considerations. Firstly, our research focused on the analysis of length of stay, survival, and readmission at the level of individual co-morbidities rather than a broader assessment of general co-morbidity status. Secondly, this approach allowed for a more detailed examination of specific health conditions and facilitated comparisons with findings from prior research that adopted a similar methodology. However, it is crucial to recognise that in any future research aimed at optimising the decision-making process regarding patients' admission to hospitals or virtual wards, an exploration of the discriminatory power of established co-morbidity indexes such as Charlson or Elixhauser would be highly beneficial.

With focus on the length of stay which is the key aspect for the target of increasing hospital bed provision by NHS in the UK (28), this retrospective longitudinal study of a virtual ward through a whole calendar year, provides evidence of shorter length of stay in hospital for patients sent to virtual wards compared to hospital inpatients. Virtual ward leads to a reduction in length of stay from 5.96 days to 2.89 days (a reduction of 3.07 days) on average, one of the shortest in the current literature as described above. The reduction in length of stay is achieved without an increase in the mortality of the virtual ward patients, although higher readmission rates and lower survival for virtual ward patients readmitted to hospital was found. Patients with no heart failure or CKD had shorter length of stay compared to the rest of the cohort.

The reduction in length of stay is an average 3.07 days for patients cared for by the virtual ward in 2022. Considering this cost was based on 366 virtual wards patients, this equates to 1123.62 hospital bed days freed in a year (3.07 x 366). This means that for a hospital with 100% occupancy (therefore providing 365 hospital bed days per bed) and 40 virtual wards beds at the capacity found in this study (24%), the virtual ward effect is an increase in the hospital capacity of 3.08 hospital beds per day (1123.62/365). The virtual ward saving of 3.08 hospital beds are around 1/9 of a whole typical 28 bedded ward. Therefore, 360 virtual ward beds are necessary to replace 28 hospital beds. The provision of 360 virtual ward would be an extensive undertaking and assumes that within the Trust there are patients who could appropriately be admitted to the 360 virtual ward beds. However, increasing the capacity to use the 40 virtual ward beds and reducing the time from hospital admission to virtual ward admission, can reduce the costs of a freed day hospital bed. Given the current virtual ward organisation, it is therefore unlikely that the virtual ward could

For peer review only - http://bmjopen.bmj.com/site/about/guidelines.xhtml

replace a whole hospital ward using the virtual ward model as it was studied in this paper. Nor is it more cost effective than traditional inpatient care. The original funding award agreed for the virtual ward was £4.4 million and would allow for a virtual ward of 160 virtual beds. That funding on the basis of this study would provide the replacement for approximately 12.5 hospital beds. But if the throughput of the virtual ward were to be doubled, then the virtual ward could replace almost a whole inpatient ward. The virtual ward would have 160 beds and would be staffed by 50 nurses and 28 additional HCA / ancillary staff – significantly greater than the requirement of the equivalent inpatient ward (35 WTE nurses and 12 WTE HCA/ancillary).

If the total cost could have covered the potential 14600 bed days from 40 virtual ward beds, than the cost for a virtual bed per day was around £72, a cost comparable to others reported in literature (i.e. €88 for virtual wards designed for severe chronic respiratory diseases (24)). However, the actual cost was of £299 for each of the 3508 bed days. In total, the cost for a patient in-hospital was £3194 on average, and that for a virtual ward patient (including the time spent in hospital) was £4336 on average. This is in contrast with some studies that have reported savings up to €8000 from virtual wards, although the studies were of not high quality (3, 7), and not comparable to current UK NHS provision.

Whilst the virtual ward is shown to be an effective way to reduce hospital stay, it requires more carers and more cost than a traditional hospital stay. To be cost-effective the virtual ward would need to double its throughput without altering the standard of care or type of patients cared for. At that level, the service would be cost effective, but would still not provide a saving on traditional inpatient care. Virtual ward care has more readmissions and looks after patients with fewer comorbidities. To provide a saving on traditional care it would need to aspire to tripling throughput. Without further efficiency in the virtual ward provision (29), it remains a clinically effective way to care for patients but not a cost effective or efficient way to care for patients.

This retrospective longitudinal study has several limitations. First, the relatively limited sample size combined with the manual matching process certainly introduces the potential for selection bias, as the matching may not comprehensively account for all relevant variables. In addition, including variations in matching ratios and the inability to find suitable matches for some virtual ward patients, may have introduced variability in the comparison groups. For the cost analysis, while efforts are made to estimate costs associated with virtual ward and hospital admissions, variations in staffing levels, assumptions made in cost calculations and the unavailability of estimates for the second half of a patient's hospital stay introduce uncertainties into the accuracy of the cost estimates, and therefore, limit the completeness and generalisability of the cost findings.

Acknowledgments

We thank the WWL financial and healthcare teams for supporting this work, and all the WWL patients, which data contributed to the findings of this work.

Contributors

All authors conceived and designed the study. AU and MF collected and screened the data. AJ and LS analysed and interpreted the data. AJ and LS drafted the first version of the manuscript. All authors critically revised the manuscript for important intellectual content. MF and LS supervised the study. AU and MF had full access to all the data in the study and take responsibility for the integrity of the data and the accuracy of the data analysis. MF is the guarantor. The corresponding author attests that all listed authors meet authorship criteria and that no others meeting the criteria have been omitted.

For peer review only - http://bmjopen.bmj.com/site/about/guidelines.xhtml

Funding

LS is supported by the NIHR Health and Social Care Delivery Research (HSDR) programme (NIHR134540) and North West Cancer Research UK (LI2021SEDDA). The views expressed are those of the author(s) and not necessarily those of the NIHR or the Department of Health and Social Care.

Competing interests

All authors have completed the ICMJE uniform disclosure form at www.icmje.org/disclosure-of-interest/ and declare: LS's institution received consultancies fees from WWL for the submitted work. MF is the Associate Medical Director at Wrightington, Wigan and Leigh Teaching Hospitals. AU and AJ declare no conflict of interest.

Ethical approval

Ethics application for use of anonymised secondary data has been approved by the Faculty of Health and Medicine Ethics Committee on the 13th of June 2023 (FHM REC Reference: FHM-2023-3699-DataOnly-3. Title: Evaluation of virtual wards in a medium size hospital in UK).

Data sharing

Data are available on reasonable request. All data were anonymised and data sharing followed the Trust Governance protocols and procedures. Identifiable patient-level data from this project are not available to the public. Data are available on reasonable request from martin.farrier@wwl.nhs.uk.

The lead author (LS) affirms that the manuscript is an honest, accurate, and transparent account of the study reported; no important aspects of the study have been omitted.

Dissemination to participants and related patient and public communities

Dissemination of this study to patients, clinicians, researchers, and the public will be done through Lancaster University website and social media; WWL website and social media; through the researchers' private social media accounts including LinkedIn and Twitter; and through a dedicated press release with a plain language summary, including direct engagements and interview with the media, locally and globally. We will share the study's information and conclusions with NHS boards, clinicians, patients and parliamentary groups. A public lecture will be organised in Wigan by Lancaster University.

Provenance and peer review

Not commissioned; externally peer reviewed.

REFERENCES

1. Best J. The virtual wards aiming to ease hospital pressures. Bmj-Brit Med J. 2022;378.

For peer review only - http://bmjopen.bmj.com/site/about/guidelines.xhtml

2. Alboksmaty A, Beaney T, Elkin S, Clarke JM, Darzi A, Aylin P, et al. Effectiveness and safety of pulse oximetry in remote patient monitoring of patients with COVID-19: a systematic review. Lancet Digit Health. 2022;4(4).
3. Norman G, Bennett P, Vardy ERLC. Virtual wards: a rapid evidence synthesis and implications for the care of older people. Age Ageing. 2023;52(1).
4. Eines TF, Storm M, Gronvik CKU. Interprofessional collaboration in a community virtual ward: A focus group study. Scand J Caring Sci. 2023.
5. Creavin ST, Garg M, Hay AD. Impact of remote vital sign monitoring on health outcomes in acute respiratory infection and exacerbation of chronic respiratory conditions: systematic review and meta-analysis. Erj Open Res. 2023;9(2).
6. Kanyimo E. Using virtual wards and long-term conditions management network to improve practice and performance. Bmj Open Qual. 2022;11(4).
7. Chauhan U, McAlister FA. Comparison of Mortality and Hospital Readmissions Among Patients Receiving Virtual Ward Transitional Care vs Usual Postdischarge Care A Systematic Review and Meta-analysis. Jama Netw Open. 2022;5(6).
8. Lasserson D, Cooksley T. Virtual wards: urgent care policy must follow the evidence. Bmj-Brit Med J. 2023;380.
9. Rockwood K, Theou O. Using the Clinical Frailty Scale in Allocating Scarce Health Care Resources. Can Geriatr J. 2020;23(3):210-5.
10. Dhalla IA, O'Brien T, Morra D, Thorpe KE, Wong BM, Mehta R, et al. Effect of a Postdischarge Virtual Ward on Readmission or Death for High-Risk Patients A Randomized Clinical Trial. Jama-J Am Med Assoc. 2014;312(13):1305-12.
11. Rue H, Martino S, Chopin N. Approximate Bayesian inference for latent Gaussian models by using integrated nested Laplace approximations. Journal of the Royal Statistical Society: Series B (Statistical Methodology). 2009;71(2):319-92.
12. NICE. Safe staffing for nursing in adult inpatient wards in acute hospitals: National Institute for Health Care and Excellence; 2014 [Available from: https://www.nice.org.uk/guidance/sg1/resources/illustration-of-process-pdf-11945197.
13. Guest JF, Keating T, Gould D, Wigglesworth N. Modelling the annual NHS costs and outcomes attributable to healthcare-associated infections in England. BMJ Open. 2020;10(1):e033367.
14. Moody S, Lamb T, Jackson E, Beech A, Malik N, Johnson L, et al. Assessment and management of secondary bacterial infections complicating Mpox (Monkeypox) using a telemedicine service. A prospective cohort study. Int J Std Aids. 2023;34(7):434-8.
15. Ward TJC, Mavilakandy A, Danns J, Tsaknis G, Reddy RV. Outcomes from a virtual ward delivering oxygen at home for patients recovering from COVID-19: a real world observational study. Clin Med. 2022;22(3):197-202.
16. Uminski K, Komenda P, Whitlock R, Ferguson T, Nadurak S, Hochheim L, et al. Effect of post-discharge virtual wards on improving outcomes in heart failure and non heart failure populations: A systematic review and meta-analysis. Plos One. 2018;13(4).
17. Majoor K, Vorselaars ADM. Home monitoring of coronavirus disease 2019 patients in different phases of disease. Curr Opin Pulm Med. 2023;29(4):293-301.
18. Mackay I, France M, McAuley D, Wing S, Wheeldon M, Britton S, et al. COVID-19 (Omicron strain) hospital admissions from a virtual ward - who required further care? Influenza Other Resp. 2023;17(3).
19. Ko SQ, Chua CMS, Koh SH, Lim YW, Shorey S. Experiences of patients and their caregivers in a Virtual Ward in Singapore: A descriptive qualitative study. Int J Med Inform. 2023;177.
20. van Ramshorst J, Duffels M, de Boer SPM, Bos-Schaap A, Drexhage O, Walburg S, et al. Connected care for endocarditis and heart failure patients: a hospital-at-home programme. Neth Heart J. 2022;30(6):319-27.
21. Kirkcaldy A, Jack BA, Cope LC. Health care professionals' perceptions of a community based 'virtual ward' medicines management service: A qualitative study. Res Soc Admin Pharm. 2018;14(1):69-75.

For peer review only - http://bmjopen.bmj.com/site/about/guidelines.xhtml

22. Tan MWJ, Arciga MGA, Arba'in JB, Towle RM, Lim SF, Tang WH, et al. Outcomes of a "hospital at home" programme for the supervised home recovery of COVID-19 patients in Singapore. Proc Singap Healthc. 2023;32.
23. O'malley EJ, Hansjee S, Abdel-Hadi B, Kendrick E, Lok S. A Covid -19 Virtual Ward Model: A Preliminary Retrospective Clinical Evaluation From a UK District General Hospital. J Prim Care Communit. 2022;13.
24. Cushen B, Madden A, Long D, Whelan Y, O'Brien ME, Carroll D, et al. Integrating hospital and community care: using a community virtual ward model to deliver combined specialist and generalist care to patients with severe chronic respiratory disease in their homes. Irish J Med Sci. 2022;191(2):615-21.
25. Lewis C, O'Caoimh R, Patton D, O'Connor T, Moore Z, Nugent LE. Utilisation of a Suite of Screening Tools to Determine Adverse Healthcare Outcomes in an Older Frail Population Admitted to a Community Virtual Ward. Int J Env Res Pub He. 2021;18(11).
26. Charlson ME, Carrozzino D, Guidi J, Patierno C. Charlson Comorbidity Index: A Critical Review of Clinimetric Properties. Psychother Psychosom. 2022;91(1):8-35.
27. Darvall JN, Boonstra T, Norman J, Murphy D, Bailey M, Iwashyna TJ, et al. Retrospective frailty determination in critical illness from a review of the intensive care unit clinical record. Anaesth Intensive Care. 2019;47(4):343-8.
28. Hayward E. NHS virutal wards available for 10,000 patients by September. The Times. 2023.
29. van der Lande ACMH, Treskes RW, Beeres SLMA, Schalij MJ. Continuum of Care: Positioning of the Virtual Hospital. Front Cardiovasc Med. 2022;8.

For peer review only

For peer review only - http://bmjopen.bmj.com/site/about/guidelines.xhtml

Length of stay and economical sustainability of virtual ward care in a medium sized hospital of the UK. A retrospective longitudinal study

Abdollah Jalilian[1,*], Luigi Sedda[1,*,$], Alison Unsworth[2] and Martin Farrier[2,#]

Supplementary information

Supplemental Table S1. Comparison of matching health indicators between all 366 patients admitted to virtual wards and the 318 virtual ward patients who were matched with patients admitted exclusively to the hospital. Based on t-tests for equality of means and chi-square tests for equality of proportions, no significant differences were found between the two groups at the significance level of 5%.

	Matched virtual ward patients (318)	All virtual ward patients (318+48)
Average CFS score	3.97	3.99
Average age	67.7	67.97
Average length of stay in virtual wards (from virtual wards onboarding to virtual wards offboarding)	9.79 days	9.64 days
Total number of virtual ward days	3114	3508
Number (percentage) of female patients	174 (54.7%)	203 (55.5%)
Number (percentage) of patients with diabetes	47 (14.8%)	54 (14.6%)
Number (percentage) of patients with hypertension	103 (32.4%)	125 (34.2%)
Number (percentage) of patients with chronic heart disease	36 (11.3%)	45 (12.3%)
Number (percentage) of patients with chronic kidney disease	2 (0.6%)	7 (1.9%)
Number (percentage) of patients with chronic obstructive pulmonary disease	81 (25.5%)	90 (24.6%)
Number (percentage) of patients with asthma	8 (2.5%)	14 (3.8%)

For peer review only - http://bmjopen.bmj.com/site/about/guidelines.xhtml

Supplemental Table S2. Costs for the provision of virtual ward for the calendar year of 2022.

Item	Whole Time Equivalent	Cost £
Nurses (band 5 and above)	12.5	520,032
Healthcare Assistant / Ancillary / Admin (Band 4 and below)	6.8	189,538
Pharmacists	2	122,239
Consultants	0.5	46,709
Corporate non clinical	1.45	148,165
Hardware		5,874
Travel		935
Medical Equipment		13,529
Additional Costs		4,129
Total		1,051,150

For peer review only

STROBE checklist compliance

Strobe Statement

Title and abstract

1a and b Compliant

Introduction

2 and 3 Compliant

Methods

4 and 5 Compliant

6 As far as is possible this is explained in the text. Matching criteria are included.

7 Compliant

8 Compliant. The sources of data are the same for each group.

9 Compliant. This is addressed in the text.

10 Compliant. This is addressed in the text. The study size was "all patients"

11Compliant. This is addressed in the text.

12 Compliant. This is addressed in the text

Participants

13 Compliant. There are 2 stages, the study period (2022) and a check on whether the patient is alive at 30th April 2021. All patients are considered at both stages. There is no additional value in a flow diagram.

14 Compliant. This is addressed in the text.

15 Compliant.

16 Compliant.

17 Compliant.

Discussion

18 Compliant.

19 Compliant.

20 Compliant.

For peer review only

21 Compliant.

Other information

22 DOI completed for each of the authors. No funding has been received for this work.

For peer review only
